# Risk Index of Regional Infection Expansion of COVID-19: Moving Direction Entropy Study Using Mobility Data and Its Application to Tokyo

**DOI:** 10.2196/57742

**Published:** 2024-08-21

**Authors:** Yukio Ohsawa, Yi Sun, Kaira Sekiguchi, Sae Kondo, Tomohide Maekawa, Morihito Takita, Tetsuya Tanimoto, Masahiro Kami

**Affiliations:** 1 School of Engineering The University of Tokyo Tokyo Japan; 2 School of Engineering Mie University Tsu Japan; 3 RCAST The University of Tokyo Tokyo Japan; 4 Trust Architecture, Inc Tokyo Japan; 5 Navitas Clinic Tokyo Japan; 6 Medical Governance Research Institute Tokyo Japan

**Keywords:** suppressing the spread of infection, index for risk assessment, local regions, diversity of mobility, mobility data, moving direction entropy, MDE, social network model, COVID-19, influenza, sexually transmitted diseases

## Abstract

**Background:**

Policies, such as stay home, bubbling, and stay with your community, recommending that individuals reduce contact with diverse communities, including families and schools, have been introduced to mitigate the spread of the COVID-19 pandemic. However, these policies are violated if individuals from various communities gather, which is a latent risk in a real society where people move among various unreported communities.

**Objective:**

We aimed to create a physical index to assess the possibility of contact between individuals from diverse communities, which serves as an indicator of the potential risk of SARS-CoV-2 spread when considered and combined with existing indices.

**Methods:**

Moving direction entropy (MDE), which quantifies the diversity of moving directions of individuals in each local region, is proposed as an index to evaluate a region’s risk of contact of individuals from diverse communities. MDE was computed for each inland municipality in Tokyo using mobility data collected from smartphones before and during the COVID-19 pandemic. To validate the hypothesis that the impact of intercommunity contact on infection expansion becomes larger for a virus with larger infectivity, we compared the correlations of the expansion of infectious diseases with indices, including MDE and the densities of supermarkets, restaurants, etc. In addition, we analyzed the temporal changes in MDE in municipalities.

**Results:**

This study had 4 important findings. First, the MDE values for local regions showed significant invariance between different periods according to the Spearman rank correlation coefficient (>0.9). Second, MDE was found to correlate with the rate of infection cases of COVID-19 among local populations in 53 inland regions (average of 0.76 during the period of expansion). The density of restaurants had a similar correlation with COVID-19. The correlation between MDE and the rate of infection was smaller for influenza than for COVID-19, and tended to be even smaller for sexually transmitted diseases (order of infectivity). These findings support the hypothesis. Third, the spread of COVID-19 was accelerated in regions with high-rank MDE values compared to those with high-rank restaurant densities during and after the period of the governmental declaration of emergency (*P*<.001). Fourth, the MDE values tended to be high and increased during the pandemic period in regions where influx or daytime movement was present. A possible explanation for the third and fourth findings is that policymakers and living people have been overlooking MDE.

**Conclusions:**

We recommend monitoring the regional values of MDE to reduce the risk of infection spread. To aid in this monitoring, we present a method to create a heatmap of MDE values, thereby drawing public attention to behaviors that facilitate contact between communities during a highly infectious disease pandemic.

## Introduction

During the COVID-19 pandemic, policies, including stay-at-home orders and social distancing measures, had been introduced worldwide [1] as measures for terminating synchronized mobility. Although these measures are known to delay or suppress COVID-19 infections during their application period [2,3], several adverse effects have been reported, including increases in infections of specific viruses in patients staying at home or in hospitals [4,5]. Moreover, mental stress, economic inactivity, domestic violence, and variations in the types of crimes have been reported [6-10], especially in urban regions with high population density, which alone cannot always explain the phenomenon of infection spread [11].

Thus, other measures have been developed, such as “stay with your community” (SWYC) [12], which was introduced in Japan in December 2020 [13,14] as a measure to suppress infection expansion. SWYC refers to sustaining fewer contacts with individuals from unfamiliar communities than with those from familiar communities, where community refers to a group of people whose members frequently come into an infectious connection in daily life. The spread of infection is estimated to be significantly magnified if individuals violate the SWYC measure. Other governmental measures studied previously can also be regarded as approaches to sustaining fewer contacts with unfamiliar individuals [3,4,15]. These can be regarded as solutions to suppress the spread of infection via intercommunity contact resulting from globalization, urbanization, and mobility [16-18]. The approach can be considered as a mild strategy in the sense that it is less stringent than stay-at-home orders because each individual can meet community members who may be classmates or colleagues in the workplace and than the “bubbling” strategy where each individual should live within one’s local community called a bubble by cutting off intercommunity contact [15]. Constraint to sustain fewer contacts with individuals from unfamiliar communities, which we refer to as constraint on intercommunity contact, is inferred to be violated if individuals from various communities (offices, schools, conferences, etc) gather in a place narrow enough for them to contact each other within a sufficiently close distance of the infection. However, an index to quantify the extent of nonadherence to the constraint on intercommunity contact, which can be measured physically, is missing.

In this study, we developed a new index that can be physically quantified and used to assess the risk of infection spread in each local region. The term “local region” refers to a defined range of space, such as a municipality (city, ward, or village, excluding islands), as a part of a prefecture or a meshed area of a certain width (eg, 1 km^2^). In the case of necessity, we have clarified the corresponding geographical area. The index we propose integrates mobility and intercommunity connectivity, which are key factors contributing to the spread of infection in each local region. In terms of mobility, the correlation between human movement and infections has been analyzed [19,20], and infections involving viruses transmitted by mosquitoes have been found to be partially driven by human mobility [21]. Specifically, the radius of gyration has been used to measure activities that affect the spread of infection in urban areas [22,23]. On the other hand, it has been discovered that a local region embracing the diversity of mobility patterns can show various responses to changes in an environment where various communities interact [24-26]. Controlling such complex interactions through interventions focused on popular venues, where people gather from various communities, may reduce both the peak infection rate and the total infected population, while retaining high social activity levels [27].

Partial differential equations have been used to predict the spread of infection via inter- and intraregional interactions [28]. The silent index for 5 industrial domains in a country has been shown to correlate with COVID-19 infection cases by a lag of weeks [29] but is not applicable to risk estimation for a finer mesh of 100 m^2^ or 1 km^2^ or by finer temporal resolution, which is desired for proposing ways to live in each local region [14]. In this study, we highlight the diversity of the moving directions of people, to which moving individuals may not pay attention, as a risk factor for local regions breaching the constraint on intercommunity contact due to increased opportunities for individuals from different communities to meet in a single location.

In this study, we show the correlation involving a new index, moving direction entropy (MDE), which represents the diversity of the moving directions of individuals in each local region and the spread of infection. We assumed that the direction of an individual’s movement reflects one’s interest in moving to or from the community. Based on this assumption and using a data set on the movements of individuals, we expected MDE to represent the extent to which the constraint on intercommunity contact would be violated. Thus, we computed the correlation between the rate of infection in the population and MDE in a local region to evaluate the utility of MDE as a measure of the risk of infection expansion. The correlations between MDE and other infectious diseases, such as influenza and sexually transmitted diseases (STDs), were also evaluated. We found that MDE is strongly correlated with the spread of COVID-19 and obtained evidence supporting our hypothesis. Furthermore, we computed the temporal variation in MDE values for local regions in Tokyo to clarify the changes in the nonadherent activities of individuals in urban settings. Finally, we propose the application of MDE as a new risk measure for suppressing infection expansion and suggest the use of an urban risk map.

## Methods

### Hypothesis: Virus Infectivity and Impact of Intercommunity Contact

To consider situations that violate the constraint on intercommunity contact, we set hypothesis A, which involves the tendencies of different infectious diseases during the expansion of infection. Hypothesis A is as follows: if the infectivity of a virus, defined as the number of other individuals that an infective individual can infect, is larger, the impact of intercommunity contact on infection expansion becomes larger.

**Figure 1:**
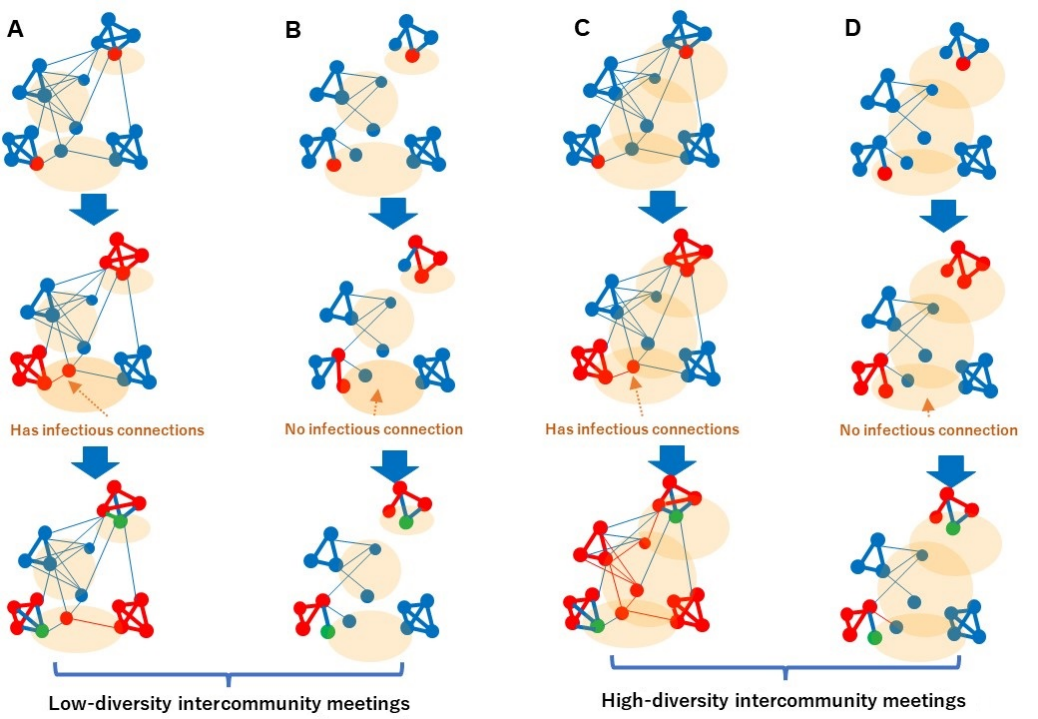
Viral infections spread across 2 types of networks. A and C: Strong intercommunity bridges (eg, COVID-19); B and D: weak intercommunity bridges (eg, sexually transmitted diseases). Influenza is positioned as intercommunity bridges of intermediate strength. Meeting places are represented by orange shadows (C and D).

The intuitive meaning of hypothesis A is illustrated in Figure 1, using a social network model. To consider the structural features of contacts, the spread of infection has been modeled using social networks [30-36]. To understand the influence of the above constraint violation on the spread of infection, it is desirable to control contacts among individuals by setting node degrees for the strategic manipulation of social networks, as in a previous report [34]. For example, SWYC was discovered in a previous report [12] using scale-free networks, among other models, modified by reflecting spatiotemporal constraints in a real society on the degrees of nodes in the network. STDs have been analyzed using social network models [35] and were also modeled using scale-free networks [36].

In Figure 1, the edges represent infectious contacts among individuals and the thick edges represent communities. Multiple communities are densely interconnected via thin edges in Figures 1A and 1C forming a large connected subgraph that includes intercommunity contacts represented by thin edges, that is, less frequent than within each community represented by thick edges. Figures 1B and 1D are composed of smaller connected subgraphs than those in Figures 1A and 1C. Hypothesis A states that infection spreads widely across communities if intercommunity infectious bridges, shown by thin edges, are dense owing to the strong infectivity of the virus via individuals attending intercommunity meetings in real spaces (orange shadows in Figure 1), as shown in Figure 1B. That is, the widened intercommunity meeting places in the orange shadows, as in Figures 1C and 1D, mediate infections only if intercommunity bridges are dense, as in Figure 1C, where infectious connections exist in the meeting places.

Figures 1A and 1C correspond to highly infectious diseases, such as COVID-19, or infections in densely populated areas, such as restaurants, in urban regions. Figures 1B and 1D show diseases with low infectivity, such as STDs, that are normally transmitted via intimate contact or exceptional hubs, such as prostitutes. Therefore, the node degrees here are assumed to be lower, corresponding to low infectivity [37]. A moderately infective species, such as influenza, whose infectivity based on the basic reproduction number is lower than that of COVID-19 [38-40] or COVID-19 in less populated areas, is positioned as an intercommunity bridge of intermediate strength between Figure 1A/C and Figure 1B/D. Based on the literature, we assumed that influenza was less infectious than COVID-19, and influenza, including H7N9, H5N1, H1N1, etc, was compared with COVID-19 before February 2021. Xue et al [39] compared the Omicron variant and influenza (seasonal and 2009 H1N1) in terms of transmission rate and effective reproduction number (*Re*). Daemi et al [40] used the basic production number to compare H1N1 and COVID-19 before the appearance of Omicron.

### MDE Approach

To date, data on human mobility have been used to analyze the spread of COVID-19. In a previous report [41], the effect of social distancing was evaluated. However, it is difficult to obtain accurate results for the medium-term COVID-19 pandemic [42] because of the nonlinear relationship between the number of COVID-19 cases and human mobility. In this study, we focused on the diversity of the moving directions of people using an entropy-based index, among other physical features such as the distance between individuals. To consider the diversity of the moving directions of individuals in each local region, we developed MDE. This statistical entropic feature is obtained by aggregating movements to generate inferences about intercommunity contacts on a regional scale [43]. The limitations of this approach will be discussed later in the Limitations subsection in the Discussion section.

We define MDE in region *r* at time *t* as follows:



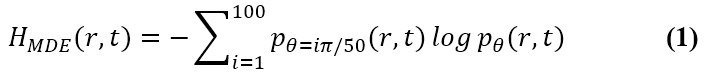



where *q* denotes the anticlockwise angle of the moving direction and 0 [rad] for the north. *q* is discretized by segmenting 2*p* into 100 segments (*p*/50 each). *p_q_* (*r*, *t*) is the probability that an individual moves in the [*q*, *q*+*p*/50] direction in region *r* at time *t*.

This definition diverts information entropy, established in information science, to consider the diversity of the collected information in order to model the behavior of individuals. Thus far, the entropy of individual trajectories has been used to study the relevance of mobility diversity to social behavior, socioeconomic indicators, and spatial attractiveness [44-46]. In the traditional mathematics of information entropy, the sensor error does not affect the ordinal results of MDE for local regions. The value of *H_ERR_*(*r*, *t*), an error function of a normal distribution with a standard deviation *σ* representing the sensing error of the moving direction, is constant as long as *σ* is constant. The value of *H*_MDE_(*r*, *t*) obtained from the sensor data is the sum of *H_ERR_*(*r*, *t*) and the true value of *H*_MDE_(*r*, *t*) because *H*_MDE_(*r*, *t*) is the convolution of *H*_MDE_(*r*, *t*) and *H_ERR_*(*r*, *t*). We did not include error bars in the analysis and discussion because we evaluated the ranking of MDE values, including *H*_ERR_(*r*, *t*), which is constant.

We applied Equation (1) to used data set 1, setting *r* to each municipality for the experiments in the Results section, and meshes (100 m^2^ or 1 km^2^) for creating the maps in the Conclusions. We then compared the results with the infection cases in used data set 2 by referring to the populations in used data sets 3 and 4.

In summary, we hypothesized that the effect of intercommunity contact is expected to be more significant if the infectivity of the target virus is high (as for COVID-19). Thus, the risk of intercommunity contact can be estimated by MDE. We used MDE as an index for estimating the pandemic risk of COVID-19, which is of high infectivity, based on the expectation from the hypothesis above.

### Data Sources

The collected data are summarized as presented below. Further information is provided in Multimedia Appendix 1.

#### Used Data Set 1: Point Data on Human Movements

The first data set used was point data on human movements in Tokyo provided by Agoop Corp. Simply put, location and velocity were sensed using GPS sensors embedded in smartphones.

#### Used Data Set 2: Number of Infection Cases

Data on the number of infection cases were collected for comparison with MDE values for (1) COVID-19 and (2) other infectious diseases.

#### Used Data Set 3: Population of Each Local Region

To validate hypothesis A, we used the data set for the population in Tokyo: (1) permanent habitats, (2) daytime populations, and (3) influx movements, provided as open data. These regions include 53 land segments, excluding the islands of Tokyo.

#### Used Data Set 4

Number of institutions (restaurants, supermarkets, etc) in each region of Tokyo.

### Ethical Considerations

The data on human movements in Tokyo (referred to as used data set 1) were smartphone-derived mobility data, and the identification of individual users was prevented by Agoop Corp [47]. The subjects provided their consent before data collection. The procedures for data collection and avoidance of the risk of personal identification were performed in accordance with the Japanese Act on the Protection of Personal Information [48] and the guidelines of the Location Business and Marketing Association of Japan [49]. This data set does not constitute a clinical trial or involve human subjects research, and is widely available for both commercial and academic research use upon purchase [50-52], which verifies that the use in this study is supported ethically by established use cases. The authors purchased the data for academic research use and obtained permission to use the data.

The collection of this data set was approved in the review of the internal committee of Agoop Corp for privacy protection and compliance management. The exemption of ethical review on this data collection and the approach of the present secondary analysis have been approved by the institutional review board (IRB) of the Medical Governance Research Institute (Tokyo, Japan; number: 22000031).

The secondary analysis is exempt from IRB review in accordance with the Ethical Guidelines for Medical and Health Research Involving Human Subjects of the Ministry of Health, Labour and Welfare, Japan [53]. The analyses on used data set 1 were also approved as an exemption by the Research Ethics Committee of the School of Engineering, The University of Tokyo (reference number: 24-23).

## Results

**Figure 2:**
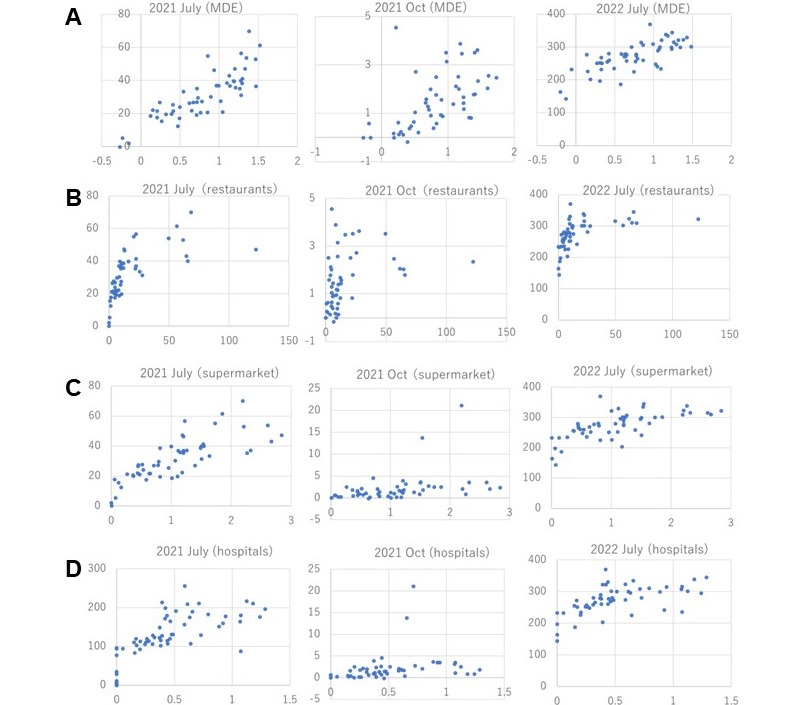
Correlation of *X* as (A) MDE and the densities (per km^2^) of (B) restaurants, (C) supermarkets, and (D) hospitals versus the rates (cases per 10,000 habitats) of infection cases (Y: COVID-19). The dots represent the values of (*X, Y*) for the 53 municipalities in Tokyo, excluding islands. Note that both values do not reflect the population but are the rates obtained by dividing the number by the width or population of each city. MDE is converted here for visualization to –log (4.6–H^MDE^). MDE: moving direction entropy.

Positive correlations were found between MDE and the rate of infection cases (number per 10,000 people) for COVID-19 in each of the 53 local regions and all municipalities, excluding islands in Tokyo Prefecture (Figure 2). First, we confirmed that the MDE values for all local regions were substantially invariant according to the Spearman rank correlation coefficient (>0.9) between the MDE values of different periods, as shown in Table 1 (generated data set 1 in Multimedia Appendix 1). We obtained the MDE values for the periods of missing data using linear completion from adjacent periods. When linear completion was impossible due to missing mobility data within 3 months of the target infection cases (April 2020), we used the MDE value of April 2021, considering the similarity of human behaviors in the same season.

**Table 1:** The invariance of moving direction entropy values quantified by the Spearman rank correlation for 53 local regions in Tokyo between different periods.

Variable^a^	December 5-11, 2019	October 10, 2020^b^	December 3-9, 2020	April 1, 2021^b^	July 26-August 9, 2021	October 18-31, 2021	April 1, 2022^b^	July 25-August 8, 2022	October 17-30, 2022
December 5-11, 2019	1.0	0.96	0.96	0.93	0.91	0.91	0.91	0.91	0.92
October 10, 2020^b^	0.96	1.0	1.0	0.96	0.94	0.93	0.92	0.92	0.92
December 3-9, 2020	0.96	1.0	1.0	0.97	0.94	0.93	0.92	0.91	0.91
April 1, 2021^b^	0.93	0.96	0.97	1.0	0.99	0.99	0.98	0.98	0.98
July 26-August 9, 2021	0.91	0.94	0.94	0.99	1.0	0.99	0.99	0.99	0.99
October 18-31, 2021	0.91	0.93	0.93	0.99	0.99	1.0	1.0	1.0	0.99
April 1, 2022^b^	0.91	0.92	0.92	0.99	0.99	1.0	1.0	1.0	1.0
July 25-August 8, 2022	0.91	0.92	0.91	0.99	0.99	1.0	1.0	1.0	1.0
October 17-30, 2022	0.92	0.92	0.91	0.98	0.99	0.99	0.99	1.0	1.0

^a^The dates show the periods when the moving direction entropy (MDE) values were computed.

^b^The MDE values for periods of missing data using linear completion from adjacent periods.

As shown in Table 2 (generated data set 1 in Multimedia Appendix 1), the value of the Spearman rank correlation coefficient between MDE and the infection cases of COVID-19 in municipalities was over 0.6 for all periods (average 0.76) of COVID-19 expansion. The correlation values were relatively low in December 2020 and October 2021, during which the correlation also decreased for other indices showing the densities (ie, the number of restaurants, supermarkets, etc per km^2^) obtained from used data set 4. Note that the values of *X* and *Y* in Figure 2 are divided by area width and population, respectively, which means that there is no effect mediated by the region size. As shown in Tables 2 and 3, the correlation between MDE and the rate of infection was smaller for influenza than for COVID-19, and tended to be even smaller for STDs.

Furthermore, the following tendencies were observed: (1) MDE and the number of restaurants were most strongly correlated with the rate of COVID-19 infection during the tested periods (Figure S1 in Multimedia Appendix 1); (2) influenza was correlated with MDE less strongly than COVID-19 but was more strongly correlated with the densities of elementary, junior, and high schools; and (3) the density of listed companies had higher correlations with STDs than with other indices.

**Figure 3:**
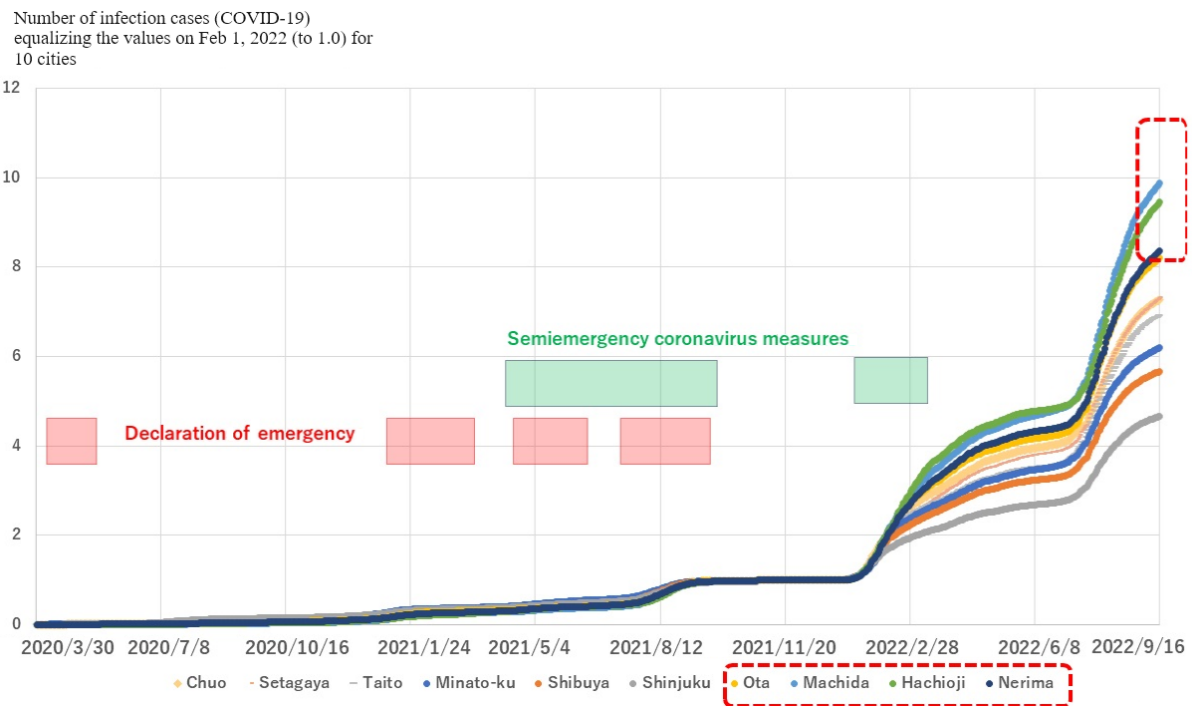
The transition of the number of infection cases for 10 randomly selected municipalities in Tokyo. Because the value is equalized in the period of the most frequent declaration of emergency (by 2021 in Figure S1 in Multimedia Appendix 1), the most radical uptrends in Ota, Machida, Hachioji, and Nerima in this figure (in the dotted frames) show the expansion after the declaration. These regions show high-rank moving direction entropy and low-rank density of restaurants (generated data set 1 in Multimedia Appendix 1). We do not show the error bars because the curves only show the raw data.

Regarding the first tendency, after periods of the governmental declaration of emergency, rapid upward trends were found in cities with high-rank MDE values and low-rank restaurant densities, as shown in Figure 3 (generated data set 2 in Multimedia Appendix 1). Local regions that did not have a high-rank density of restaurants (ranked lower than 20th) showed a significantly larger expansion if they were relatively highly ranked on MDE (within the 30th highest) than those with higher than the 20th density of restaurants (*P*<.001; generated data set 1 in Multimedia Appendix 1).

**Table 2:** Correlation of the rates of infection cases (COVID-19) and explanatory indices (moving direction entropy and the densities of institutes in local regions) quantified by the Spearman rank correlation for 53 local regions in Tokyo.

Indices			Rates of infection cases: COVID-19 for 3 years																											
			2020									2021							2022							Average			*P* value^a^	
			Before April 5		July 26		October 17		December 3		April 1			July 26		October 18		January 25			April 1		July 25							
MDE^b,c^			0.77		0.83		0.73		0.62		0.84			0.85		0.67		0.87			0.71		0.75		0.77			—^d^		
**Density (/km^2^)**																														
	Restaurants	0.74		0.86		0.77		0.65		0.82			0.85		0.55		0.83			0.76		0.78		0.76			.37			
	Supermarkets	0.74		0.81		0.77		0.66		0.75			0.83		0.58		0.80			0.77		0.73		0.74			.11			
	Elementary schools	0.65		0.83		0.69		0.54		0.74			0.80		0.60		0.76			0.73		0.74		0.71			.002			
	High schools	0.62		0.78		0.72		0.57		0.70			0.79		0.55		0.72			0.67		0.67		0.68			*<*.001			
	Junior high schools	0.66		0.81		0.72		0.56		0.73			0.80		0.63		0.73			0.73		0.72		0.71			.004			
	Train stations	0.67		0.83		0.74		0.63		0.79			0.79		0.56		0.82			0.71		0.77		0.73			.02			
	Hospitals	0.56		0.70		0.65		0.56		0.68			0.69		0.54		0.71			0.57		0.66		0.63			*<*.001			
	Listed companies	0.72		0.82		0.73		0.60		0.78			0.81		0.59		0.79			0.74		0.77		0.74			.02			
	Population	0.70		0.86		0.71		0.55		0.76			0.84		0.65		0.78			0.71		0.75		0.73			.01			

^a^*t*-test; *P* values show the significance of the superiority of MDE to other indices.

^b^MDE: moving direction entropy.

^c^The MDE values were computed on used data set 1 for each period, and the rates of cases were obtained from used data set 2 for each period of 2 weeks.

^d^Not applicable.

**Table 3:** Correlation of the rates of infection cases (sexually transmitted diseases and influenza) and explanatory indices (moving direction entropy and the densities of institutes in local regions) quantified by the Spearman rank correlation for 53 local regions in Tokyo.

Indices			STDs^a^ (December 3-17, 2019)									Influenza	
			Gonorrhea		Genital herpes		Chlamydia		Condyloma acuminatum				
MDE^b^			0.48		–0.06		0.56		–0.18		0.45		
**Density (/km^2^)**													
	Restaurants	0.53		–0.06		0.60		–0.15		0.38			
	Supermarkets	0.49		–0.08		0.57		–0.17		0.44			
	Elementary schools	0.43		–0.12		0.52		–0.22		0.43			
	High schools	0.48		–0.09		0.54		–0.18		0.51			
	Junior high schools	0.45		–0.10		0.52		–0.20		0.55			
	Train stations	0.52		–0.05		0.60		–0.14		0.34			
	Hospitals	0.45		–0.09		0.53		–0.18		0.37			
	Listed companies	0.64		0.13		0.70		0.04		0.40			
	Population	0.43		–0.13		0.51		–0.23		0.44			

^a^STD: sexually transmitted disease.

^b^MDE: moving direction entropy.

**Figure 4:**
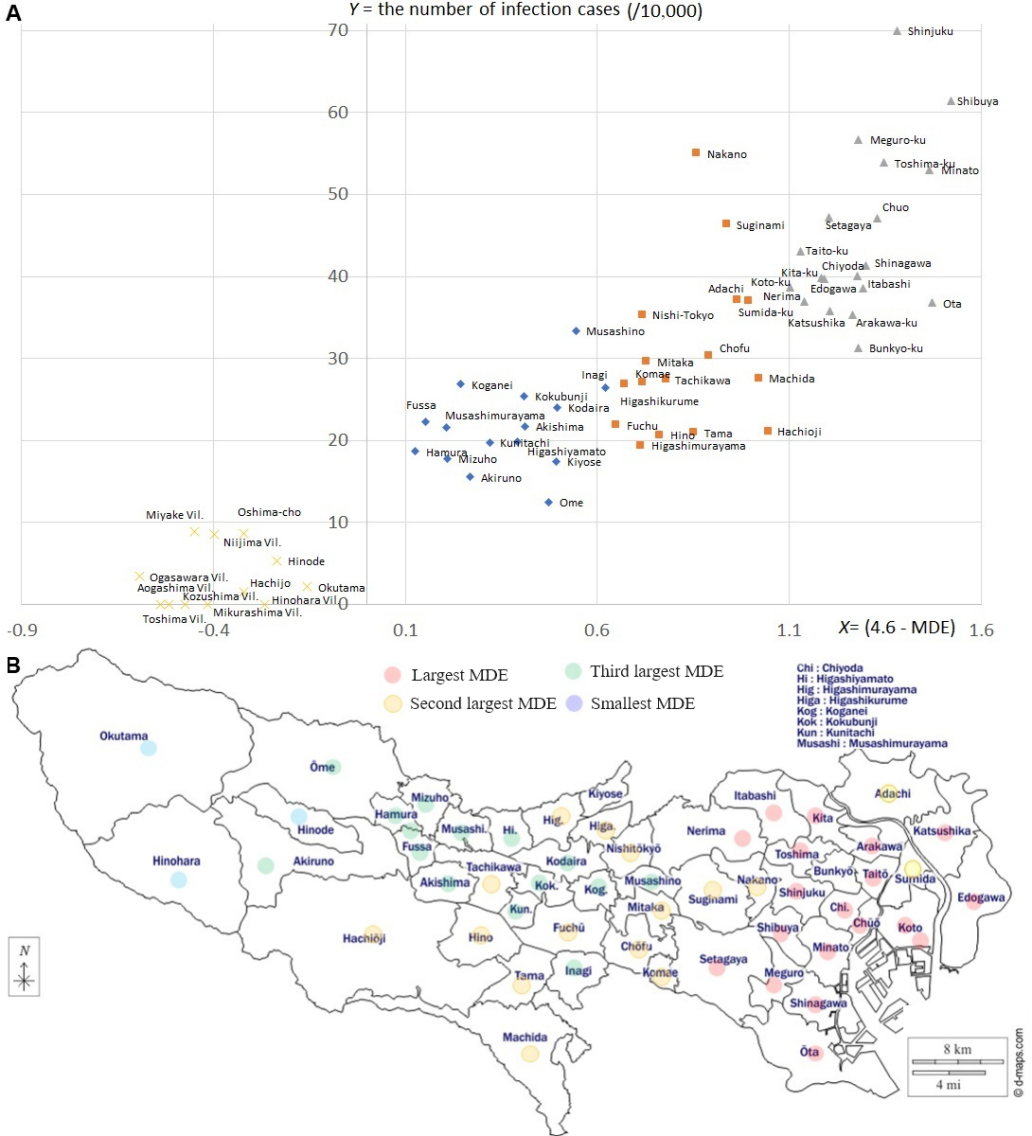
(A) Distribution of (*X, Y*) from Figure 2A in local regions. (B) Their locations in the map. The plots in A are clustered corresponding to the colored regions in B, that is, the most active part in the east, the next most active part, and the least most active part. Part A includes islands in the lowest cluster to show comparability with Okutama, Hinohara, and Hinode, which are the furthest from the central part. A free-license map (free Tokyo map) is used. MDE: moving direction entropy.

**Figure 5:**
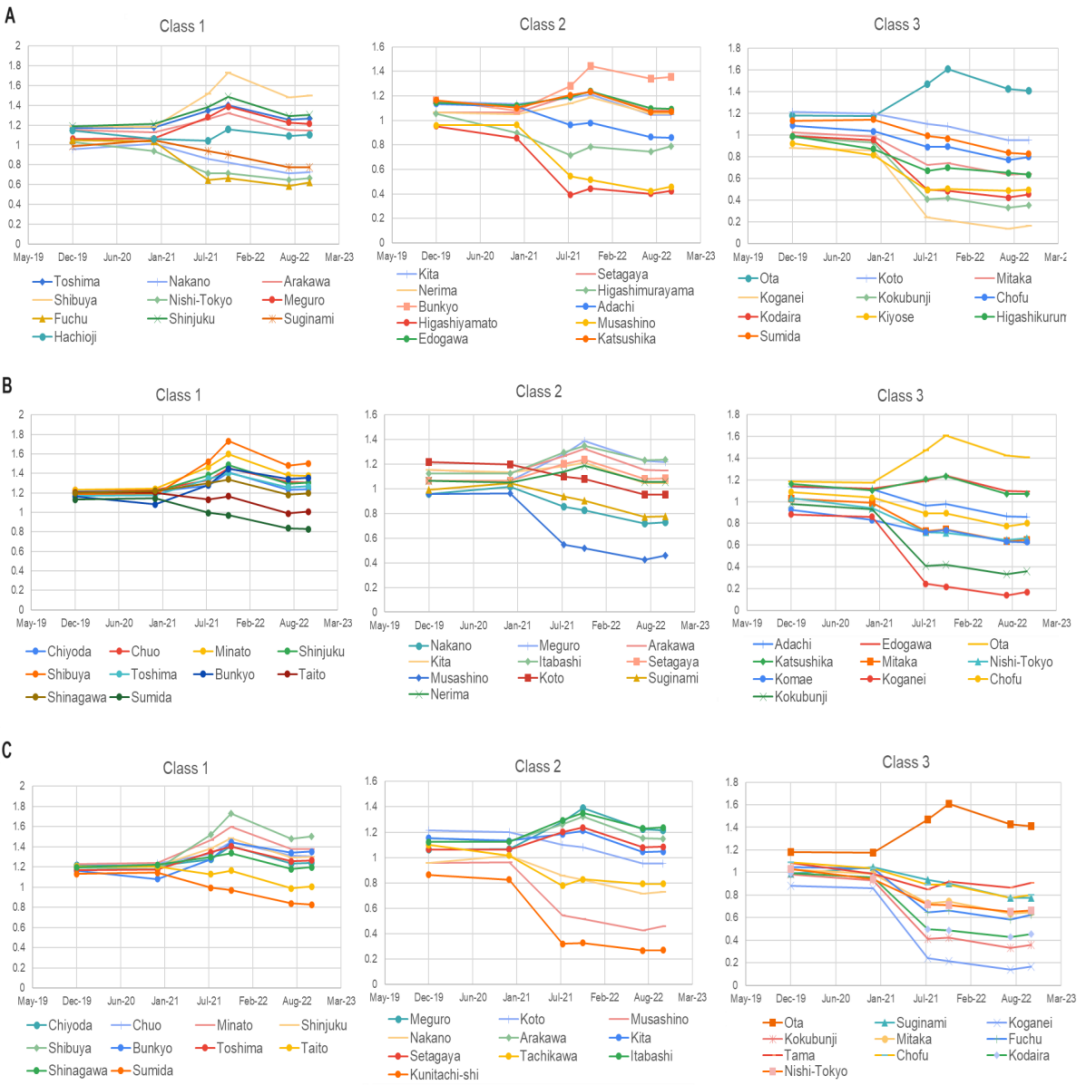
Transition of moving direction entropy (MDE) in local regions classified according to population density (A: permanent, B: daytime, C: influx flow). In parts B and C, MDE in class 1 increased during the period of COVID-19 infection expansion. Individuals in cities with high interregional activities are believed to stay careful in these periods, avoiding densely populated areas, but their movements were against their careful attitudes, according to the results here. We do not show error bars for MDE (X-axis; discussed in the Methods section).

In Figure 4A from generated data set 3 in Multimedia Appendix 1, the upper-right (lower-left) cluster includes local regions with larger (smaller) MDE and larger (smaller) numbers of infected cases. The locations of these regions are shown on the map of Tokyo in Figure 4B [54]. In Figure 5, the regions are classified based on the population densities of (1) permanent habitats, (2) daytime populations, and (3) influx movements from used data set 3.

## Discussion

### Principal Findings

The correlation of MDE with the rate of COVID-19 cases was larger than that of other factors (Table 2), although it was not significantly larger than that of restaurant density. This strong correlation provides useful information for creating government measures. That is, mobility in and to restaurants has already been restrained by governmental measures, such as the declaration of emergency, but MDE has been left without attention. As a result, a significantly rapid expansion in cities with high-rank MDE and low-rank restaurant densities was found (Figure 3). This shows the necessity of adding MDE as an index to evaluate the regional latent risk of infection spread.

Because MDE is regarded as an index representing the extent to which individuals from different communities contact each other, the relative weakness of the correlation of MDE with cases of influenza and STDs compared with COVID-19 coincides with hypothesis A, considering the infectivity with respect to Figure 1 A/C and B/D. That is, Figures 1A and 1C show that a stronger trend of intercommunity infectious contacts accelerates the spread of the virus if it is highly infective, whereas such an acceleration is not observed in Figure 1B or 1D for less infective viruses. This also coincides with Figure 4A, where the upper-right cluster corresponds to the busiest regions, including the wards in central Tokyo, as shown in Figure 4B. In contrast, the lower-left cluster in Figure 4A has the lowest MDE and the lowest number of infected cases and includes the western (low population) part of Tokyo and the islands.

The transition of MDE implies an improvement or worsening of the behavior of individuals in local regions concerning the spread of infection. The local regions in classes 1 and 2 in Figures 5A-C with large MDE values correspond to the upper cluster in Figure 4A, while those in class 3 with lower MDE values correspond to the lower cluster in Figure 4A, including the western part of Tokyo, as shown in Figure 4B, and the islands belonging to Tokyo. However, the regions with the largest densities of daytime population and influx (moving from other regions) experienced an upward trend in MDE, as in class 1 (Figures 5B and 5C), during the COVID-19 expansion period that started in the spring of 2020 and remained stable from the summer of 2020 (Figure S1) until July 2021. Such a coincidence between the expansion period and the upward trend of MDE was not obvious in Figure 5A where class 1 did not show an upward trend and class 2 did not show a more obvious downward trend than class 1. Thus, the accelerated dynamic movements of individuals fostered the interaction of communities, corresponding to an increase in MDE. In addition, individuals in the active regions were found to take fewer safe actions during risky periods, as shown by the upward trends of MDE in class 1 (Figures 5B and 5C).

We evaluated the correlation between MDE and the spread of infection, but we do not claim to have verified a causal relationship. We proposed MDE as an index for assessing the risk of infection retrospectively from the data by showing only the correlation. However, causal relationships have been published, including the abovementioned relationship [22], and this showed that an increase in COVID-19 infection reduces the range of movement and diversity, which is the opposite of the dedication of causality and its content. That is, the previous authors analyzed the causality from the spread of infection to human movements, whereas we evaluated the risk of infection spread relevant to human movements. Moreover, the previous authors showed negative causality, whereas we showed a positive correlation. Thus, the causality associated with the diversity of movements due to the spread of COVID-19 is unlikely, and the fact that a correlation was found may indicate a likelihood that a causality of COVID-19 infection spread due to the diversification of movements is present. However, there is a limitation in determining whether this is indeed a causal relationship, as it would require an interventional method to actually cause the spread of infection, which would be ethically unsuitable.

### Limitations

The results should be interpreted with consideration of some limitations. It is debatable whether mobility patterns obtained from a data set on mobility collected by smartphones may be generalized to the public [55] because smartphone users may not be representative of the changes in mobility of the population as a whole, and their representativeness may vary by location and season, as pointed out by Wellenius et al [3]. In addition, because of the privacy policy of the provided data set, we were unable to assess the characteristics of individuals using smartphones, such as age, gender, race, and income.

However, currently, data available from smartphones on the approval of owners are some of the most comprehensive and prevalent data. The use of data, such as GPS sensor data, is a standard approach in the face of these limitations in several studies, including the studies mentioned above and other reports [18,22,41], which restrict the data subjects further to Facebook users. Compared with studies using other digital methods, such as extracting geolocation data from wearable trackers [56,57] or geotagged social media posts [58], we used data from a wider range of samples. Furthermore, restricting smartphone data is beneficial for users because it enables a simple, automated, widespread, and easy-to-use method for risk assessment in the future. Thus, the fact that the results of this study were obtained from smartphone data implies their usefulness for infection risk assessments as people are expected to continue to possess smartphones in the future.

In addition, we introduced a statistical entropic feature by aggregating movements to generate inferences about dynamics at a regional scale and discarding unknown heterogeneities. This approach may not cope with the limitation pointed out in a previous report [19] that the spatial location of the infection may differ from the location of the surveillance system. In Japan, the locations of infection and residence may differ, and the number of infected persons in the area of residence needs to be surveyed. We obtained high accuracies for MDE in sensing latent risk in this study, even with this limitation. First, during the COVID-19 expansion period, people changed to living in the local regions of their residences due to the stay home policy and their cautiousness. Second, regarding the interregional movement that was still taking place, the below discussion indicates that it makes sense to observe the correlation between MDE and the number of infected people counted in residential regions.

Among mobilities within region *r* that affect the MDE of *r*, the mobility of residents inside *r* is directly related to the spread of infection. Let us consider an example scenario for comprehension. The first scenario is as follows: An essential worker, a nurse, or a carer living close to (within the same region *r*) the hospital or house of the patient they support is from a community, that is, a nurse station, a care support office, or his or her own family, which is different from the patient’s family. Similarly, a visitor to a family living close is from a different family.

On the other hand, the mobility within *r* of those from residences outside *r* also affects a part of the MDE of region *r* because this mobility reflects the diversity of the communities they come into contact with inside and outside *r*. The second scenario is as follows: A nurse or care manager, who lives in *r*′ and visits a family in *r* (*r∩r*′=ϕ), generates an intercommunity interaction because his or her community differs from the family to visit.

Thus, the mobility within *r* of individuals from both inside and outside *r* is related to the spread of infection among the residents of *r*. We additionally evaluated the influence of the mobility of those living and acting only outside *r* on the spread of infection in region *r*. The third scenario is as follows: A nurse or care manager, who lives in their own region *r*′ (r∩r′=ϕ) and interacts only within *r*′, shows an intercommunity interaction in *r*′ similar to the case of the first scenario. Such individuals are indirectly related to the infection expansion in *r* because they contact other essential workers who visit region *r*, as in the second scenario.

### Comparison With Prior Work

The point of this paper is not to present the “best” index for risk assessment, although comparisons with other indices, such as population density, have been made (Table 1). In fact, the superiority of MDE over population density was not significant (*P*=.11). Because MDE is an independent measure that is not affected by population density (MDE is probability-based), we propose combining MDE with other indices, including population density, rather than choosing a better index. Such a combinatorial use of indices is essential for risk assessment and protective policies in the future. For example, the government of a local region may state the following: “this city has low population density, a small number of supermarkets, etc, but MDE is high. So, citizens should be careful!” or “most people wear masks in this city, but MDE is high. We should consider measures other than masks and urge citizens to take vaccines.” Because MDE is only a statistical indicator for the likeliness of infection spread, based on the probability of moving direction, combining it with other indices is an essential approach for finer risk assessment. In this sense, MDE is also expected to be used as a variable to reinforce the approach of using similar mobility data [50] or multivariate machine learning [59] and further improve the accuracy of the prediction of infection cases.

From a technical perspective, mobility-based studies on the spread of infection using sensors, such as GPS in smartphones, are geographically restricted. For example, studies were limited to smartphone owners in 4 cities in northern Thailand [22], geolocated social media users in New York City [58], the city of El Pasto in Texas [60], and parks in the United States [61]. Compared with the locations in these studies, Tokyo is a large city with 14 million inhabitants. The 53 municipalities range from urban areas to mountainous areas with very small populations, including regions with various prepandemic periods (2019 for all regions in Tokyo, and even until the end of 2020 for suburbs such as Kokubunji, Kunitachi, Inagi, and Hamur; all regions had severe outbreaks in 2022), and thus, there were regions with diverse situations. Therefore, the data used in this study are suitable for our attempts to derive laws not limited to activities in particular industries or regions.

Compared with the approach of the “Three Cs” applied in Japan during the pandemic period [62,63], where individuals were forbidden to meet in a closed space, crowded place, or close-contact setting, the measure to reduce MDE can be applied to realize secure movements rather than as a way of staying in a meeting place.

However, controlling human mobility in urban transit [64,65] could not necessarily be regarded as an effective method for suppressing the spread of the COVID-19 pandemic because urban transit may not have a stronger influence on this spread than mobility in the areas of groceries and pharmacies [66]. These results coincide with those in Table 2, where the number of train stations had a weaker correlation with the number of infections than did supermarkets.

The regions have been colored independently of the population or its density because they depend only on the values of MDE obtained from the sheer probabilistic distribution of the movement directions of individuals. We should recollect the better fitness of MDE rather than the population density of these locations, as shown in Table 2, which could shed light on the controversy regarding the impact of population density [11].

### Conclusions

This study has 4 essential findings. First, the MDE values for local regions showed significant invariance between different periods from before to within the COVID-19 pandemic period. Second, MDE was found to be significantly correlated with the rate of COVID-19 infection cases among local populations in the 53 inland regions of Tokyo. The correlation between MDE and the rate of infection was smaller for influenza than for COVID-19, and tended to be even smaller for STDs (order of infectivity of the virus). This supports the hypothesis that the impact of intercommunity contact on infection expansion increases for a virus with higher infectivity. Third, the spread of COVID-19 accelerated in regions with high-rank MDE values compared to those with high-rank restaurant densities during and after the period of the governmental declaration of emergency. Fourth, the MDE values tended to be high and increased during the COVID-19 period in regions where influx or daytime movement was present.

A possible explanation for the third and fourth findings is that policymakers and living people have been overlooking risk factors corresponding to MDE, even during the governmental declaration of emergency. Furthermore, the infection-suppression effort and its effects on the sheer reduction of movements tended to weaken significantly after the relaxation of strict control measures [67]. Thus, we propose to keep monitoring MDE values and, if MDE increases, send out messages to satisfy the constraint on intercommunity contact, such as the SWYC guidelines, or be cautious when visiting a place where people move in diverse directions. However, we did not consider controlling individual movements as the main measure because it would be difficult for a single person to lead the movements of others. Thus, we regard it as the practical use of MDE to improve the social environment, such as the development of pathways and other infrastructure to unify the direction of pedestrian movement in urban areas, where possible. If individual behaviors are to be controlled, it should only be added as a supplementary message to environmental improvement approaches, such as encouraging people to wear masks in areas where there is inevitably a diversity of directions of movement or to avoid visiting places where people move in various directions. In addition, it is difficult from a research ethics perspective to analyze data on individual movements.

**Figure 6:**
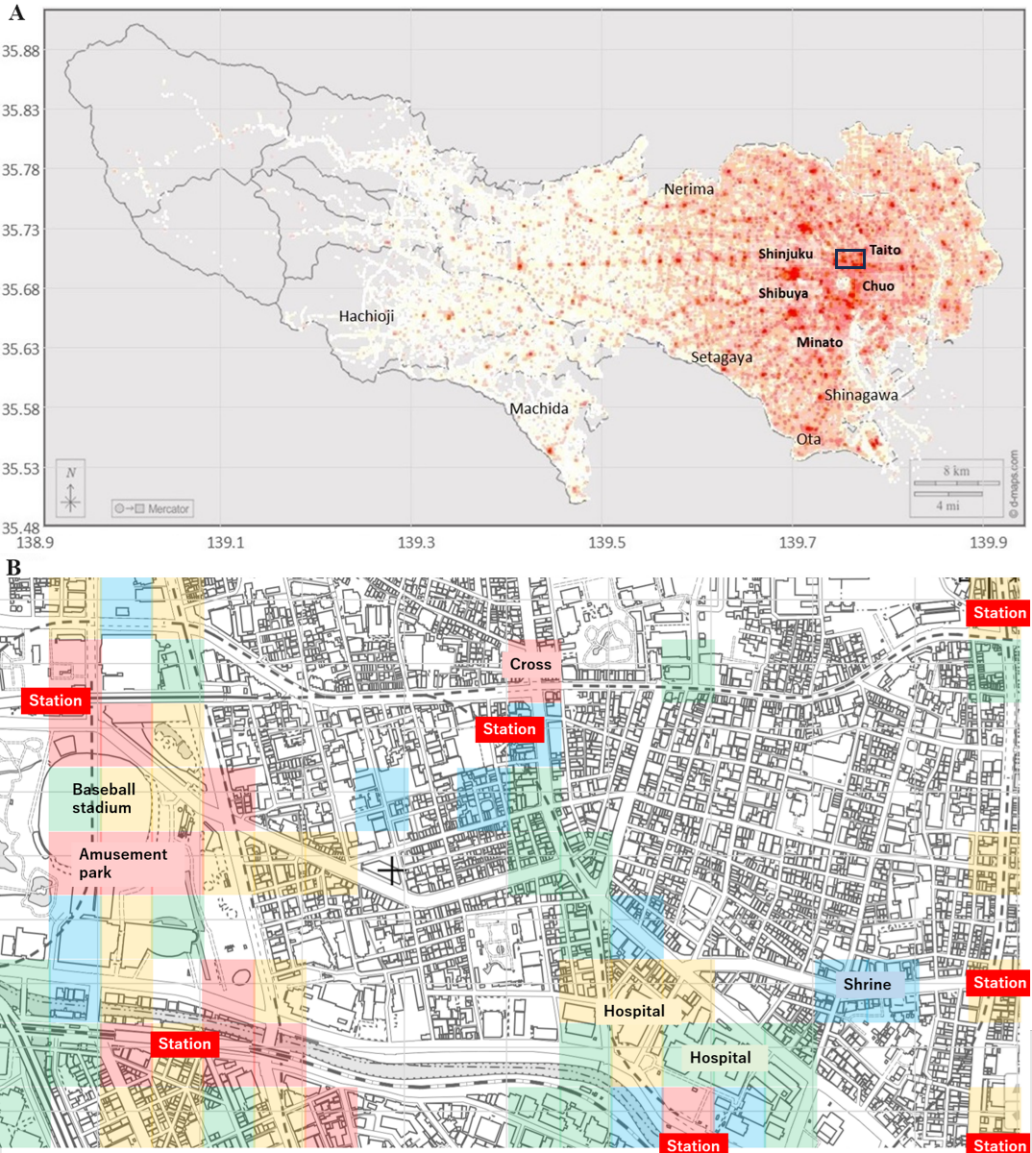
The heatmaps of moving direction entropy (MDE) for 100-m^2^ meshes in A all of Tokyo, except islands, and B a part of Bunkyo ward near the University of Tokyo. The small rectangle in A corresponds to B. Meshes are classified according to MDE values (red: top 1000 meshes in Tokyo, yellow: second top 1000 meshes, green: third top 1000 meshes, blue: fourth top 1000 meshes). All use free-license maps: A includes a free Tokyo map and B includes a blank map from the Geospatial Information Authority of Japan.

We propose the application of MDE to visualize the locations of high-risk regions to accelerate the awareness of both individuals and society as a whole regarding the risks in local regions. Individuals living or working in regions highlighted in the heatmap of MDE (Figure 6) will enhance their risk awareness, and their communication will enhance the awareness of the regional society [54,68]. In Figures 6A and 6B, 100-m^2^ meshes with the highest MDE in Tokyo are shown in dense red (1000 meshes in Tokyo with the highest MDE values), and weaker colors represent the ranks of meshes according to their MDE values. Despite canceling the effects of city size and human crowd as discussed above, the dense red areas in Figure 6A and the squares in Figure 6B clustered close to the busiest stations (A: in the central wards Shibuya, Shinjuku, etc; B: in a selected ward Bunkyo). The mesh regions with the highest MDE values do not necessarily coincide with train stations, but some coincide with supermarkets and restaurants, corresponding to the literature [66].

## Appendix

Multimedia Appendix 1 Data sets generated or used in the study.

## Abbreviations

IRB: institutional review board
MDE: moving direction entropy
STD: sexually transmitted disease
SWYC: stay with your community

## Data Availability

Research data supporting this publication and its links are available in the manuscript and Multimedia Appendix 1. The device location data in used data set 1 are not publicly available because of the contract between Agoop Corp and the authors who analyzed the data, but the data may be made available upon reasonable request if both the authors and Agoop Corp agree to data sharing. The data on infection cases (used data set 2), regional population (used data set 3), and institutions (used data set 4) are publicly available and are provided in Multimedia Appendix 1. The processed data on moving direction entropy involving used data set 1 are available in Multimedia Appendix 1.
