# Supplementary material for: Risk Index of Regional Infection Expansion of COVID-19: Moving Direction Entropy Study Using Mobility Data and Its Application to Tokyo
Source: JMIR Public Health Surveill. 2024 Aug 21;10:e57742. doi: 10.2196/57742 (PMC11375397; doi:10.2196/57742)
Supplement: Multimedia Appendix 1 [file publichealth_v10i1e57742_app1.docx]

## **Multimedia Appendix 1.** Data sets generated or used in the study

#### Generated Datasets 1-3: available as separate files linked below:

<https://drive.google.com/drive/folders/1qiAeK-SxqiabvdXYkkG3ekOXxuT7wvFe?usp=drive_link>

Generated Dataset 1: Data obtained on the correlation of indices (MDEs and densities of institutes covered in the Used Dataset 4) with the number of infection cases for diseases

Generated Dataset 2: The trends of infection cases in local regions

Generated Dataset 3: MDE values and number of infected cases in local regions.

#### Used data set 1: Human mobility

Data on human movement in Tokyo were provided by Agoop Inc. Simply put, the location and velocity were collected using a GPS sensor embedded in the smartphone. For example, 615,000 unique smartphone users were monitored at 31 × 10^6^ logs per month in August 2020. The availability of data is not open here because it is a purchased dataset from Agoop Inc. Owing to the terms and conditions considered, the data were confidential. The specification of this dataset is summarized on the confidential specification material of Agoop point data.

The obtained entropy values are presented in Generated Dataset 1, and all personal information was eliminated by statistical computation. The movement directions were included in this dataset, which enabled the computation of Eq.(1). Information included was as follows:

Daily ID of a user: ID to identify the user, valid for one day, which was changed at 0:00 every day. Allocated on an app-by-app basis. If multiple applications were on the same smartphone, then different IDs were assigned. This part did *not* include personal information of participants and was not used in this study.

Time: Year, month, day, hour, and minute when the app acquired location information: time zone for logs in Japan, Japan Standard Time (JST). The data analyzed in this study were follows corresponding to Figure S1.

1. December 3 through December 10, 2019
2. December 3 through December 10, 2020
3. July 26 through August 8, 2021
4. October 18 through October 31, 2021
5. July 25 through August 8, 2022
6. October 17 through October 30, 2022

Location: Latitude and longitude represented by six digits after the decimal point, and address by the code and the name of the prefecture and the city: if the smartphone was set to use positioning information from Wi-Fi or mobile base stations etc., this information was taken into account in addition to the GPS.

Moving velocity: Calculated by the position and time between two points and by the change in the frequency of radio waves received by the terminal from the satellite.

Moving direction: Measured using a magnetic sensor on the smartphone. If the direction of movement cannot be obtained from the smartphone, the estimated direction is shown. North as 0, and clockwise to 359.999. 0-3 decimal (degrees).

#### Used data set 2: The numbers of cases of infectious diseases

Data on the number of the following infection cases were collected for comparison with MDE values, for the periods illustrated in Figure S1.

1. COVID-19 before May 2023: The infection cases obtained from tests in hospitals and other medical institutes were sent to the Tokyo Metropolitan Government (TMG). On 26 September 2022, the government moved the data on infection cases in municipalities in Tokyo to [1]. to the following URL and stopped updating it.
2. Other infectious diseases: The infection cases obtained by tests in selected (fixed-point) hospitals were sent to the government of the TMG for all diseases in the “class five” as shown in the data of [2]. Dataset (b) included (1) weekly data on the number of Influenza, and RS infection cases, and (2) monthly data on the number of STDs, including Herpes, Chlamydia, and Condyloma Acuminata. Cases of infection with these diseases were adjusted for using (c).

(c) Because the cases of these diseases were counted for selected hospitals, the number of cases was adjusted by the factor *l*(*d*, *r*)/*r*(*d*, *r*) (>1), where *l*(*d*, *r*) and *r*(*d*, *r*) are the numbers of all the medical institutes and the selected medical institutes where disease *d* was tested in local region *r*. To estimate *r*(*d*, *r*), reference [3] was used as the most up-to-date information about fixed-point medical institutions, including their affiliations and municipalities corresponding to local regions.

COVID-19 switched from May 2023 to counting the number of positive cases at selected fixed-point medical institutions in Japan. To maintain the coherence of the data collection conditions for COVID-19 throughout the target period of analysis, we used only the data in (a). Thus, the adjustment in (c) was applied only for the following diseases: influenza (institutions dealing with pediatric, internal medicine, core, or pseudo-diseases) and sexually transmitted diseases (institutions dealing with STDs, core, or pseudo-diseases). To estimate *l*(*d*, *r*), the number of hospitals conducting either antigen (quantitative or qualitative) tests in each region *r* was obtained from [4]. For STDs, the results of a search for 'sexually transmitted diseases & <test or treatment>' (in Japanese) were counted separately for each local region as in reference [5].

#### Used data set 3: Population of each local region

To validate Hypothesis A, we used the newest datasets (a), (b), and (c) below for the population of Tokyo published in 2022, provided as open data by TMG. The regions below refer to the 53 land segments in Tokyo, excluding the islands (See Figure 6A to appear later).

1. Permanent habitants: Table 1 of reference [6]
2. Daytime population, counting the number of individuals in daytime, supposed to be active people (business workers, students, shopping customers, etc.): Table 2 of reference [6]
3. Influx-flow population, counting people moving from other local regions: Table 4 of reference [6]

#### Used data set 4: The number of institutes in each region

The number of institutes in each region of Tokyo was obtained from Apamanshop.com (a private estate company) [7]. These data are included in the worksheet “Original counts” in Generated Dataset 1.


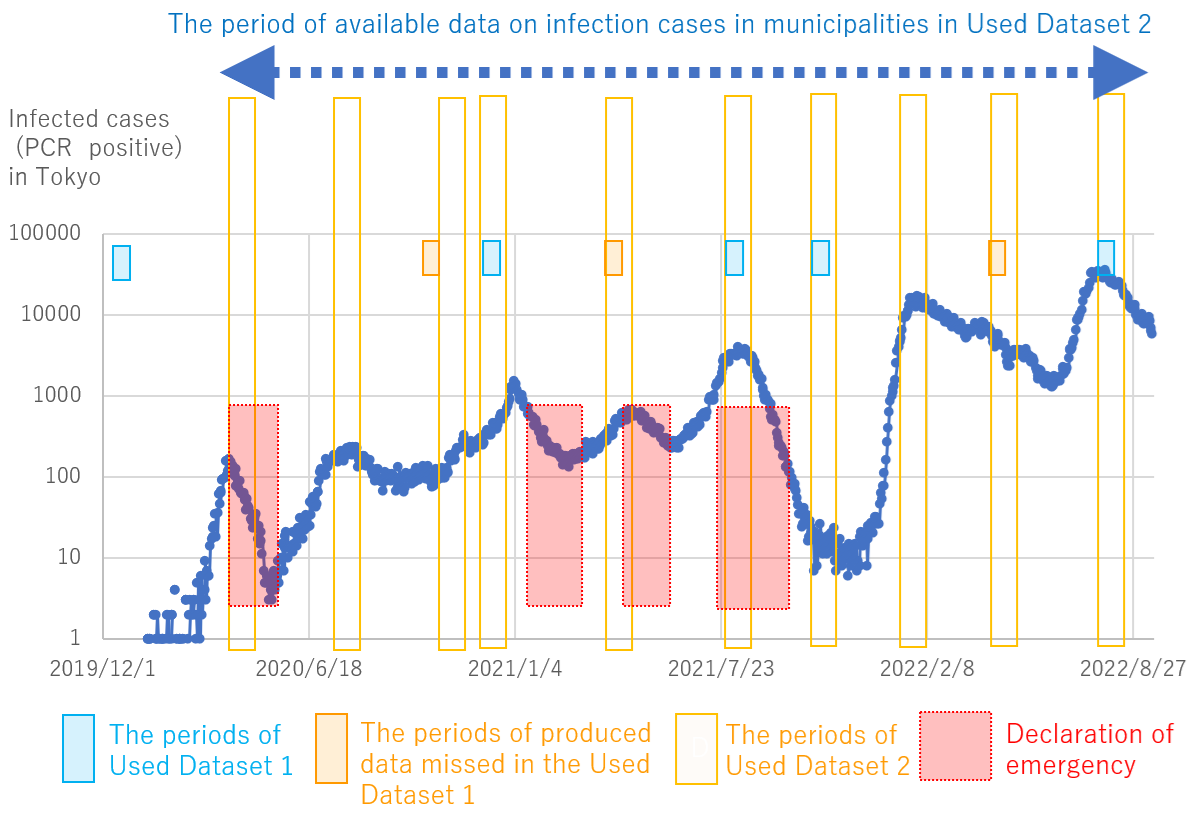


Figure S1. The transition of the number of infection cases (semi-log), the periods corresponding to the used datasets, and the declarations of emergency.

References

1. Tokyo Metropolitan Government. Number of positive cases of novel coronavirus infection by ward, city, town, and village, 2022. "https://spec.api.metro.tokyo.lg.jp/spec/t000010d0000000085-2215a7dcc2ff9f2a535063a2d4d42ece-0

2. Tokyo Metropolitan Infectious Disease Surveillance Center, Epidemiological Surveillance of Infectious Diseases, Accessed 2023. "https://survey.tmiph.metro.tokyo.lg.jp/epidinfo/epimenu.do

3. Tokyo Metropolitan Government. Tokyo Metropolitan government, List of fixed-point medical institutions for investigation of infectious diseases, 2021. https://idsc.tmiph.metro.tokyo.lg.jp/assets/year/2021/2021-3.pdf

4 Tokyo Metropolitan Government. List of medical institutions with outpatient services, Accessed 2023. https://www.hokeniryo.metro.tokyo.lg.jp/kansen/corona_portal/soudan/hatsunetsugairai.html.

5 Tokyo Metropolitan Government. Information on medical and pharmacy functions in Tokyo. Accessed 2023. https://www.himawari.metro.tokyo.jp/qq13/qqport/tomintop/

6 Tokyo Metropolitan Government. Population of Tokyo (population by place of work or school) according to the 2020 census (in Japanese) https://www.toukei.metro.tokyo.lg.jp/tyukanj/2020/tj-20index.htm

7 Apamanshop.com, Regional information of Tokyo (in Japanese), accessed 2023. https://www.apamanshop.com/tokyo/townpage/
